# Applying trauma systems concepts to humanitarian battlefield care: a qualitative analysis of the Mosul trauma pathway

**DOI:** 10.1186/s13031-019-0249-2

**Published:** 2020-02-04

**Authors:** Kent Garber, Adam L. Kushner, Sherry M. Wren, Paul H. Wise, Paul B. Spiegel

**Affiliations:** 1 Department of Surgery, University of California, Los Angeles, CA USA; 2 Center for Humanitarian Health, Johns Hopkins Bloomberg School of Public Health, Baltimore, MD USA; 3 Surgeons OverSeas, New York, NY USA; 4 Department of Surgery, Stanford University, Palo Alto, CA USA; 5 Department of Pediatrics, School of Medicine, Stanford University, Stanford, CA USA

**Keywords:** Trauma and surgical care, Trauma systems, Armed conflict, Humanitarian responses

## Abstract

**Background:**

Trauma systems have been shown to save lives in military and civilian settings, but their use by humanitarians in conflict settings has been more limited. During the Battle of Mosul (October 2016–July 2017), trauma care for injured civilians was provided through a novel approach in which humanitarian actors were organized into a trauma pathway involving echelons of care, a key component of military trauma systems. A better understanding of this approach may help inform trauma care delivery in future humanitarian responses in conflicts.

**Methodology:**

A qualitative study design was used to examine the Mosul civilian trauma response. From August–December 2017, in-depth semi-structured interviews were conducted with stakeholders (*n* = 54) representing nearly two dozen organizations that directly participated in or had first-hand knowledge of the response. Source document reviews were also conducted. Responses were analyzed in accordance with a published framework on civilian battlefield trauma systems, focusing on whether the response functioned as an integrated trauma system. Opportunities for improvement were identified.

**Results:**

The Mosul civilian trauma pathway was implemented as a chain of care for civilian casualties with three successive echelons (trauma stabilization points, field hospitals, and referral hospitals). Coordinated by the World Health Organization, it comprised a variety of actors, including non-governmental organizations, civilian institutions, and at least one private medical company. Stakeholders generally felt that this approach improved access to trauma care for civilians injured near the frontlines compared to what would have been available. Several trauma systems elements such as transportation, data collection, field coordination, and post-operative rehabilitative care might have been further developed to support a more integrated system.

**Conclusions:**

The Mosul trauma pathway evolved to address critical gaps in trauma care during the Battle of Mosul. It adapted the concept of echelons of care from western military practice to push humanitarian actors closer to the frontlines and improve access to care for injured civilians. Although efforts were made to incorporate some of the integrative components (e.g. evidence-based pre-hospital care, transportation, and data collection) that have enabled recent achievements by military trauma systems, many of these proved difficult to implement in the Mosul context. Further discussion and research are needed to determine how trauma systems insights can be adapted in future humanitarian responses given resource, logistical, and security constraints, as well as to clarify the responsibilities of various actors.

## Background

Beginning in October 2016, the Iraqi army, supported by the Kurdish Peshmerga and a U.S.-led international coalition, launched an intensive campaign to retake Mosul, once Iraq’s second largest city, from the militant group the Islamic State, which had captured the city and much of northern Iraq and western Syria in 2014. The campaign lasted nearly nine months and became arguably the largest urban siege since World War II. Nearly one million people were displaced, and thousands killed, by the time the battle ended in July 2017 [1, 2].

**Fig. 1:**
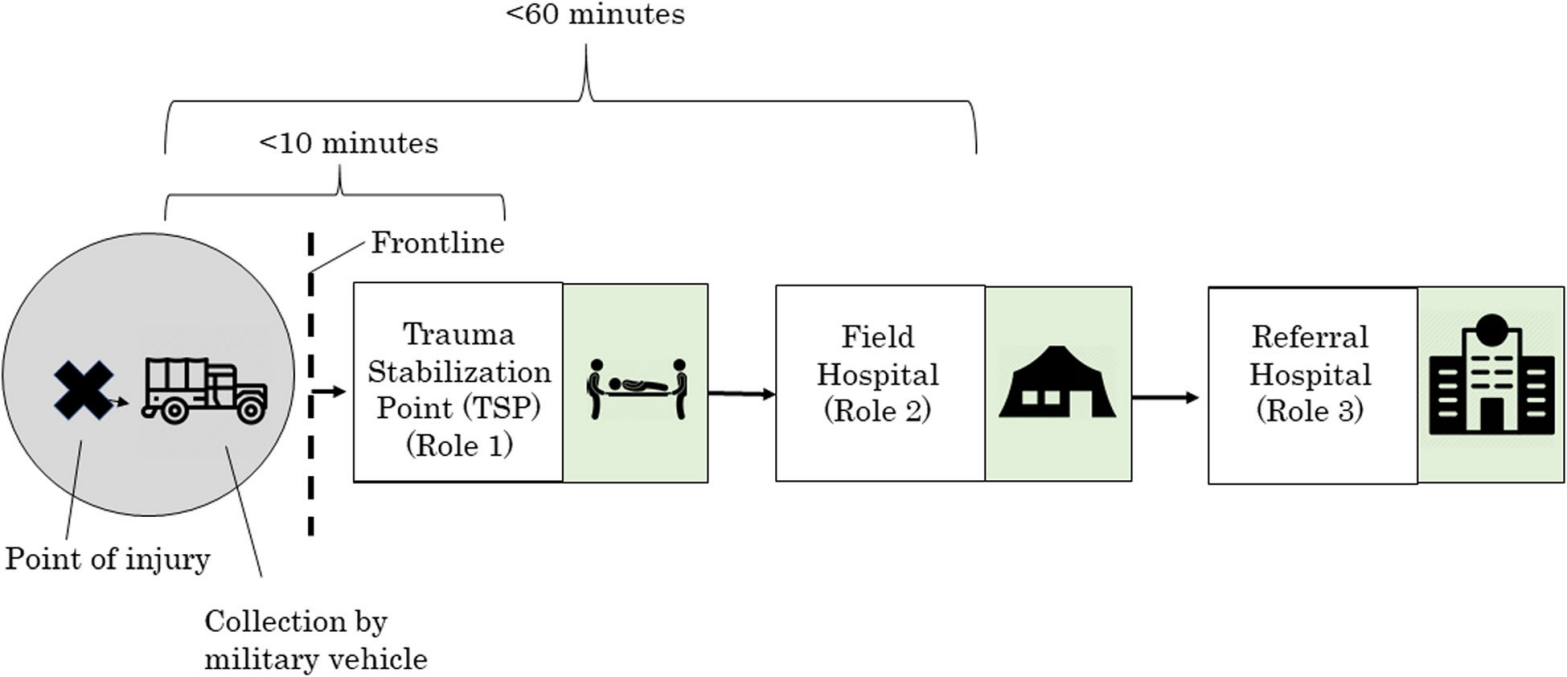
Schematic Representation of Mosul Civilian Trauma Pathway

As fighting unfolded, severe gaps in trauma care for wounded civilians emerged. Humanitarian planners, led by the World Health Organization (WHO), responded by coordinating what became a novel trauma response pathway designed to improve access to trauma and surgical care. This pathway drew upon the concept of “echelons of care” used by the North Atlantic Treaty Organization (NATO) and other military evacuation systems, in which war-wounded are stabilized near the frontlines and, when necessary, transferred “up the chain” to higher levels of care [3]. In Mosul, three levels, or echelons, of care were ultimately implemented for civilians: Trauma stabilization points (TSPs), run by medical non-governmental organizations (NGOs), were situated within 10–15 min of the frontline; field hospitals were established within roughly an hour of the point of the injury; and referral hospitals for more complex injuries were designated further away from the theatre (Fig. 1).

In many ways, this pathway represented a marked departure from “business as usual” for humanitarian trauma care in wartime. Although echelons of care are well-described in war surgery literature, they are most commonly associated with western militaries, having been deployed in military responses in Vietnam, Israel, and the Falkland Islands in the 1970s and 1980s through Afghanistan and Iraq more recently [4, 5]. These military evacuation chains provided first aid near the point of injury, transport of the critically wounded, and surgical care for combatants and, to varying degrees, injured civilians. Humanitarian actors, by contrast, have historically been constrained by resource, security, and logistical challenges and have not organized formal, military-style trauma evacuation pathways [6–10]. As the International Committee for the Red Cross (ICRC) noted, “echelons for the management of war wounded do not always exist in a civilian or humanitarian context”; rather humanitarians often work at a single site, at variable distances from the frontlines, and have been dependent upon the war-wounded getting to them by whatever means possible [11]. Even when echelons do exist, they are often rudimentary: In the 1980s, for example, the ICRC supported a series of “first aid posts” and field hospitals in Afghanistan and along the Afghanistan-Pakistan border, but it took patients 6–7 h, and at times more than a day, to reach one of the hospitals [7, 8]. Moreover, on principle, many humanitarian organizations feel strongly that care at or near the frontlines is -- and should remain -- the responsibility of professional militaries, not humanitarians, in accordance with the Geneva Conventions [12, 13].

Yet recent experiences in Iraq and elsewhere have shown that humanitarian agencies are actively reassessing, and seeking to improve, how they deliver trauma and surgical care [14, 15]. These efforts began with natural disaster responses, reflecting the fallout from the 2011 Haiti earthquake response that was widely criticized as slow, fragmented, and poorly coordinated [16]. Agencies are now re-examining trauma care in war, spurred in part by growing lessons from military battlefield trauma systems over the past two decades. In the 2000s, the U.S.-led international coalition in Iraq and Afghanistan made massive investments in battlefield trauma systems that have been credited with a marked reduction in servicemember fatality rates compared to previous armed conflicts [17, 18]. Critically, these systems feature not only multiple echelons of care (from point of injury to complex rehabilitative care), but also integrative components such as communication, transportation, data collection, and clinical practice guidelines that enabled a continuum of timely, quality care for the gravely injured [3, 5]. Many of these elements have been credited with saving lives, including reduced times between injury and definitive care (often through the use of air evacuation to limit the time between injury and definitive care to less than one hour); better tactical pre-hospital care that prioritized hemorrhage control including tourniquet use, resuscitation with blood products, and hypothermia management; sustained en-route care during transportation; and real-time use of data to improve care delivery [5, 19, 20]. In sum, the combination of improved data collection and analysis, clinical practice guidelines, and real-time clinical governance have enabled such achievements.

Given the novel application of military-style echelons of care to the Mosul humanitarian trauma response, as well as the growing interest from humanitarians to strengthen trauma care in conflict settings, there is a need to better understand what was done in Mosul and to examine how trauma systems insights were manifest in this approach and how they might be improved in the future. Accordingly, the purpose of this study is to analyze the Mosul trauma response through a trauma systems lens, drawing upon a published framework for civilian battlefield trauma systems [21]. This framework outlined a schema featuring multiple levels of care, with providers and activities designated at each level (Table 1); it also specifies six supportive or integrative components: coordination, communication, transportation, health information systems, education and training, and research. Applying this framework, the study aims to assess whether the Mosul trauma pathway functioned as an integrated system and to identify areas that could be strengthened, context-permitting, in future responses.

**Table 1:**
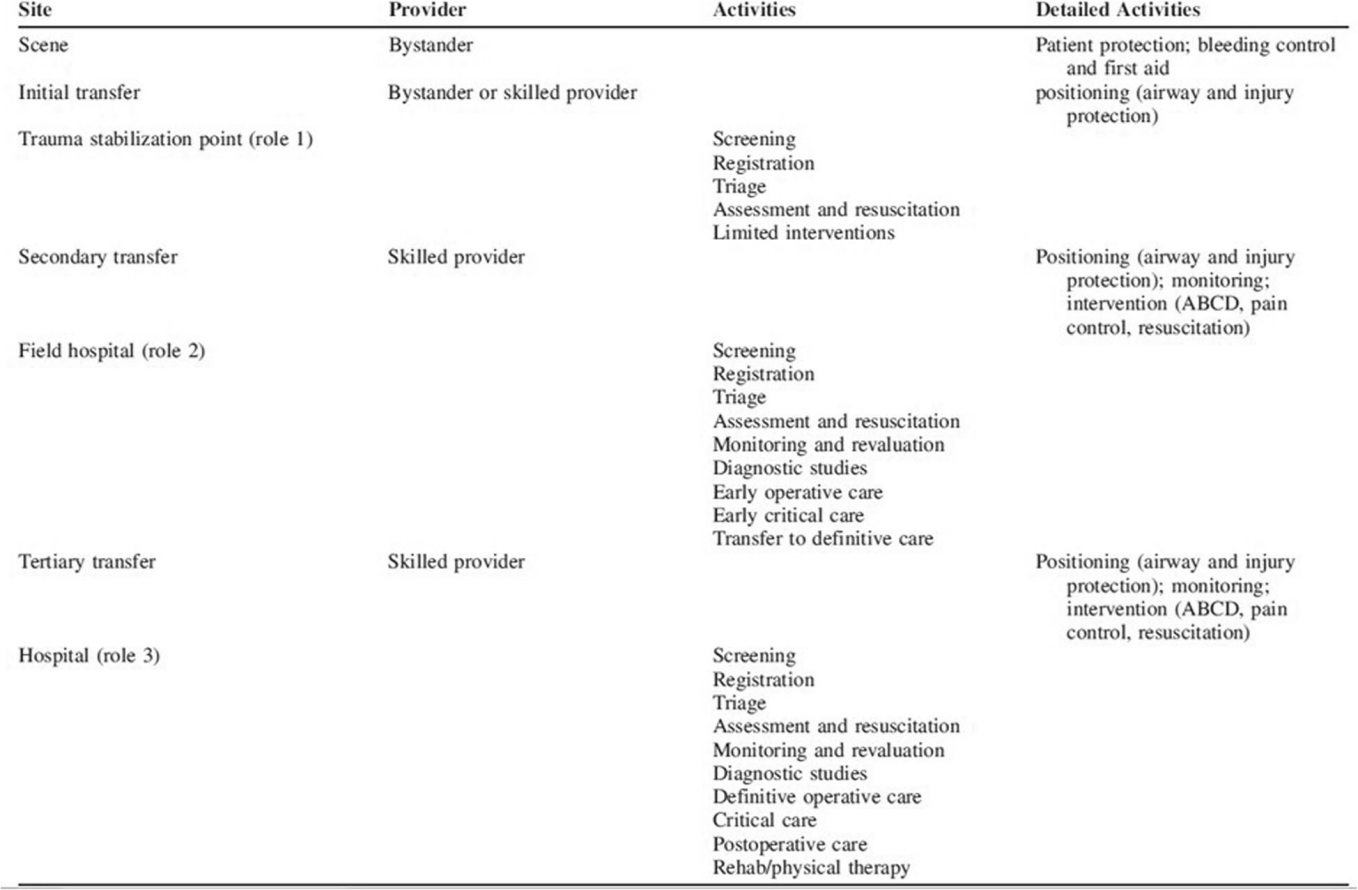
A Proposed Framework for Civilian Battlefield Trauma Systems

## Methodology

### Design and study population

A case study methodology was developed using qualitative semi-structured interviews and source document review to examine the Mosul civilian trauma response. Key organizations in the Mosul response were identified through public documents, discussions with WHO and implementing partners, and chain-referral sampling, whereby identified stakeholders suggested other relevant contacts. Individuals were purposively selected based upon their direct participation in or knowledge of the trauma response. A total of 54 interviews were conducted, including representatives from WHO, the United States Office of Foreign Disaster Assistance (OFDA), European Civil Protection and Humanitarian Aid Operations (ECHO), United Nations (UN) Office for the Coordination of Humanitarian Affairs Civil-Military coordination (OCHA CivMil), United Nations Population Fund (UNFPA), International Organization for Migration (IOM), United Nations High Commissioner for Refugees (UNHCR), Ninewah Department of Health (DoH), Samaritan’s Purse, Aspen Medical, NYC Medics, Global Response Management (GRM), CADUS, Médecins Sans Frontières (MSF), International Committee of the Red Cross (ICRC), Handicap International, Emergency Hospital in Erbil, and the U.S. military. A full listing is provided in Additional file 1. IRB exemption was granted by the Johns Hopkins Bloomberg School of Public Health IRB committee.

### Data collection

Interviews were conducted from July through December 2017. Subjects who were physically present and available during the study team’s visits to Iraq or Geneva in September 2017 were interviewed in person. All other interviews were conducted virtually by Skype. Interviews were typically conducted jointly by multiple members of the study team. Interview domains and questions were developed in advance and based upon a literature review of published studies on civilian and military trauma systems, as well as humanitarian responses in conflict settings. The key domains covered in the interviews are provided in Additional file 2. Reflecting the sensitive nature of the discussions, interviews were conducted on the agreement that responses would be attributable to the organization but not the individual, unless otherwise specified. Interviewees participated voluntarily following a formal request for interview from the study team. Interviews typically lasted 30–90 min and were recorded and transcribed or captured with detailed notes. All interviews were conducted in English.

### Document review

Interviews were supplemented by an extensive document review, including situation reports, meeting notes, planning documents, and needs assessments produced for the response by the participating organizations, as well as relevant academic literature and news reports. These included documents from the planning phase of the Mosul response, starting in summer of 2016, through conclusion of formal fighting in summer 2017. Documents were either supplied directly to the study team by interviewees or obtained through online searches. A listing of the documents reviewed is provided in Additional file 3. As with the interviews, these were analyzed against the referenced framework, and relevant information extracted in accordance with the specified domains.

### Data analysis

Interview responses and documents were analyzed against a published civilian battlefield trauma system framework [21], focusing on activities at different levels of care as well as the integrative system components (coordination, communication, transportation, health information system, education and training, and research). Transcripts and notes were used to categorize organizations by type (humanitarian, government, etc.) and role (TSP, field hospital, etc.) and coded to identify key themes based upon the framework. Findings were synthesized primarily by two authors and discussed collectively with the larger group for agreement.

### Funding

Funding for this study was provided through an independent, unrestricted grant from the United States Agency for International Development (USAID). The findings do not necessarily represent the views of USAID or the U.S. government.

## Results

### Key trauma actors

The Mosul trauma pathway encompassed a variety of actors, including NGOs, UN agencies, local civilian agencies, military forces, and one private medical company. Actors participated in one or sometimes multiple echelons of care, reflecting their respective capacities, interest, and experience. Some were present for the entire response, whereas others participated for only a portion of it. Several groups, including NYC Medics, Samaritan’s Purse, and Aspen, were supported materially by WHO, which in turn received funding from the U.S. government (OFDA), the European Union (ECHO), and the UN Central Emergency Response (CERF) Fund. Others were supported by separate donors (e.g. IOM was supported by the UK’s Department for International Development (DFID) or independent contributions (e.g. MSF). A list of the key trauma actors, with their designation and associated role in the trauma response, is provided in Table 2.

**Table 2:** Key actors in the Mosul trauma pathway for civilians

Type	Name	Role
NGO	NYC Medics	TSP provider, Coordination
	Academy of Emergency Medicine/Global Response Management	TSP provider
	Cadus	TSP provider
	Samaritan’s Purse	Field hospital
	MSF-OCB	Field hospital, Rehabilitation hospital
	MSF-OCG	Referral hospital
	MSF-OCP	Referral hospital
	Handicap International	Post-operative care and rehabilitation
UN agency	WHO	Coordination
	UN OCHA CivMil	Coordination
	IOM	Field Hospital
	UNFPA	Obstetrics units at Aspen field hospitals
Civilian	Emergency Hospital, Erbil	Referral hospital
	West Emergency Hospital, Erbil	Referral hospital
	Al-Shaikan Hospital, Duhok	Referral hospital
	Ninewah Department of Health	TSP
Other humanitarian organization	International Committee for Red Cross	Mobile surgical unit, staffing and rehabilitation at referral hospitals
	Qatari Red Crescent	Field hospital (with IOM)
Private company	Aspen	Field hospitals
Military	Iraqi military	Transportation, TSPs
	U.S.-led coalition	Multiple

Note: This list focuses on organizational roles in the Mosul trauma pathway and does not capture other health-related activities that organizations may have been performing, or trauma services provided by other actors. *OCB* Operational Center Beligum; *OCP* Operational Center Paris; *OCG* Operational Center Geneva.

### Levels of care and activities

**Fig. 2:**
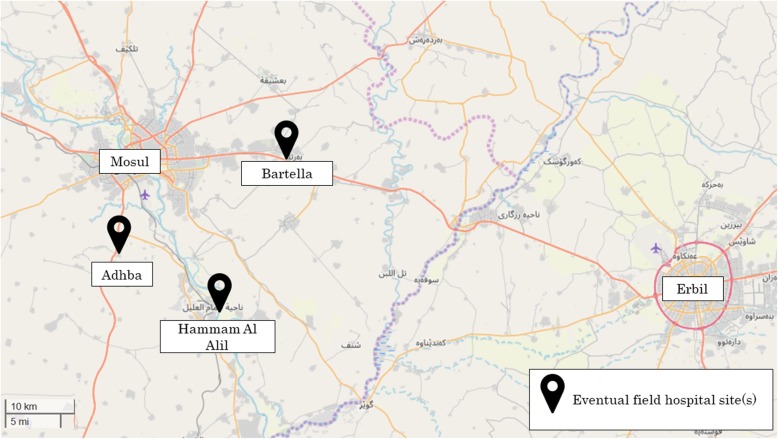
Map of Key Field Hospital Sites during the Battle of Mosul

The need for a coordinated trauma response developed in late 2016, as the frontline moved away from Iraqi Kurdistan and closer to Mosul. Options for frontline stabilization and surgery for civilians were increasingly limited (Fig. 2), as most hospitals in and around Mosul were non-functioning or lacked supplies, the Iraqi and Kurdish military had few trained combat medics, and the U.S.-led coalition, although having deployed some medical units, adopted medical rules of engagement that prioritized care for soldiers and sharply limited care of civilians. Although many casualties in the first months of fighting had been sent to Erbil (the capital of Iraqi Kurdistan), by late 2016 border crossings became increasingly difficult. Meanwhile, a handful of non-governmental actors had arrived to provide frontline medical care, but many were informally organized, had limited medical credentials, and in some cases carried weapons and engaged in hostilities.

As the gaps in professional trauma care became apparent, WHO, supported by the U.S. and EU governments, appealed to NGOs and other groups for assistance. Over the next few months, several organizations responded, or ultimately agreed, to participate in a coordinated evacuation pathway organized by WHO consisting of different echelons of care (as shown above in Fig. 1). This pathway functioned mainly during the second phase of the Mosul offensive, in West Mosul, which lasted from February–July 2017. Each echelon had a different set of activities or responsibilities, as described below:

#### TSPs

TSP teams provided stabilization and resuscitation care, with the goal of hemorrhage control and stabilizing critically ill patients near the frontlines and initiating transport to field hospitals within 10–15 min. TSPs were located within 5 km of the frontline and were intended to be mobile. They closely followed Iraqi military units to facilitate access to civilian casualties, most of whom were being transported back from the frontlines via military vehicles. Although several groups informally provided TSP-style care during the first part of the Mosul offensive (November 2016–January 2017), this approach was formalized in February 2017 with the arrival of NYC Medics to coordinate the TSP response at WHO’s request. They were joined by other NGOs, including Global Response Management and Cadus.

To develop TSP practice guidelines in Mosul, WHO drew upon its Emergency Medical Team standards for natural disasters [22]. These included hemorrhage control with appropriate use of tourniquets, airway protection using opening maneuvers and airway devices, and placement of intravenous lines for fluid resuscitation. Blood products were not routinely available at the TSP level. In the field, activities varied somewhat based upon TSP capabilities. NYC Medics was staffed with physicians who were comfortable performing more invasive procedures, such as chest tube placement, even though such actions went beyond WHO guidelines. Interviews with TSP providers indicated that tourniquet placement, fluid resuscitation, and other recommended procedures were routinely performed, but data are lacking to assess appropriateness or quality.

#### Field hospitals

Field hospitals provided emergency surgery and trauma care. They were expected to receive patients transported from TSPs within 1 h of injury, but they also treated patients who arrived by other means (i.e. outside the evacuation pathway), including those with medical emergencies and outpatient needs. Most were based in temporary structures, such as large tents or trailers, although some were set up inside pre-existing buildings. Samaritan’s Purse, a faith-based NGO, opened the first field hospital in the pathway in January 2017, with WHO’s support, about 25 km east of Mosul. In February 2017, MSF-Belgium opened the first surgical facility in West Mosul. In March and April 2017, Aspen Medical, a private company, and IOM and the Qatari Red Crescent, opened additional field hospitals around West Mosul. Other actors operated or supported field hospitals further removed from the frontlines, as shown in Table 2.

Field hospitals performed a variety of emergency trauma surgeries, including laparotomies, amputations, wound debridement, and basic fracture repairs, as well as other procedures depending upon staffing. At the Aspen and Samaritan’s Purse sites, patient turnover was high, as patients were typically discharged within 48–72 h of surgery to ensure bed space for mass casualties. Some patients were discharged to internally displaced persons (IDP) camps or returned home, but follow-up and opportunities for post-operative care and rehabilitation were limited (see below). The availability of non-trauma services at these sites also varied. Respondents indicated that Aspen initially focused almost exclusively on trauma care (e.g.. did not initially accept patients with medical issues), whereas many MSF affiliates emphasized providing medical, pediatric, and non-trauma services in addition to trauma care. UNFPA supported obstetric care services (cesarean sections and vaginal deliveries) at the Aspen field hospitals.

#### Referral hospitals

Two civilian hospitals in Erbil—Emergency Hospital and West Emergency Hospital—were designated as the primary “end point” hospitals for more complex injuries, including spinal cord injuries, brain trauma, and burns. Some field hospitals, depending upon staffing, also served in a referral capacity. The IOM/QRC hospital, for example, accepted vascular injuries from other facilities, and Samaritan’s Purse accepted complex orthopedic injuries from other sites. MSF-Belgium, recognizing a gap in rehabilitative care, operated a rehabilitative hospital to care for patients with complex wounds or post-operative needs. Handicap International worked in a number of facilities and IDP camps to provide rehabilitative care.

### Integrative trauma system components

In military battlefield trauma systems, echelons of care are linked by integrative components to ensure that care is continuous, timely, and of high quality. The availability of these components in the Mosul humanitarian response is described below:

#### Coordination and communication

At the field level, coordination was undertaken by NYC Medics, which oversaw patient transfers, conducted hospital assessments, and monitored bed and service availability at different sites. As one NYC Medics member noted:

*“Part of our involvement was setting up a referral system, figuring out what was the closest hospital, where should we send patients, coordinating all those movements so that in a mass casualty patients didn’t show up at the same hospital. The referral system was disorganized when we first arrived. We had people doing capacity mapping to figure out what hospitals were capable of receiving. We were also coordinating referrals between field hospitals and between field hospitals to tertiary hospitals in Erbil.”*

Many respondents applauded NYC Medics for embracing this role and executing it almost singlehandedly throughout the response but felt that field coordination could have benefited from greater funding, staffing, and technical support from WHO.

At the strategic level, respondents cited UN OCHA CivMil, a coordinating body that facilitates dialogue between military and civilian actors, as playing a critical intermediary role between Iraqi and Coalition partners and humanitarian planners. Many felt that OCHA CivMil offered vital security and logistical support that helped protect medical workers in the field and kept military actors appraised of their presence. Providers also met via a weekly trauma working group under the auspices of the UN health cluster, the coordinating body for the Mosul humanitarian health response. Most providers said they found these meetings to be valuable for identifying operational challenges, discussing solutions and aligning responses given the number of actors involved in the response.

#### Transportation

Dozens of ambulances were procured during the response, and organizers made repeated efforts to increase the number and positioning of ambulances. However, respondents indicated that orders and shipments were often delayed due to customs issues and the need for multiple government approvals (both Kurdish and Iraqi), and the lack of ambulances was a commonly cited problem. Interviewees indicated that most ambulances were not stocked with medications or medical supplies, and trained medical personnel were often not available to accompany patients between levels of care (i.e. from TSPs to field hospitals, or from field hospitals to referral hospitals), reflecting the lack of available local medical personnel. As a result, en-route care was often limited, likely leading to some disruptions in treatment. In some cases, TSPs providers did accompany critically ill patients on the ambulance, requiring them to leave their posts. Data on transport times were not collected. Drivers were sometimes unclear about where to go, and sometimes ambulances would be commandeered by the military for other purposes, as one respondent noted:

*“There were difficulties. Sometimes ambulance drivers didn’t know where to go, sometimes ambulance drivers would go where they felt the most comfortable going. It’s a tricky landscape when you have people walking around with rifles near the TSPs, and someone with a gun telling you where [a military patient] needs to go, even though the ambulance is supposed to be used for civilian purposes.”*

Air evacuation was reportedly provided to some wounded soldiers by Iraqi and/or Coalition forces, but this option was not routinely available for civilians, according to respondents.

#### Health information systems

To standardize data reporting, WHO provided templates to field hospitals, and NYC Medics developed data collection forms for the TSPs. At the TSP level, this included data on demographics, vitals, mechanism on injury, anatomic location, triage status, time in and out, treatments received, and disposition status. At the field hospital level, data included admissions, deaths on arrival, hospital deaths, average length of stay, injury type, and type of surgeries performed. However, data collection proved challenging throughout the response. There was variability among organizations in the completeness and quality of their data reporting; data categories were sometimes not clinically relevant or were changed; and potentially useful outcomes metrics were not captured. In particular, there was no system for tracking patients from TSPs to field hospitals or from one hospital to the next, limiting conclusions about the response’s effectiveness. As one respondent noted:

*“There was no follow up on cases referred [up the chain]. The idea was that you would stabilize and refer out. The outcomes at the next level – no one has any idea.”*

Although a new data entry platform was adopted in spring 2017 to improve data collection, discussions with participants indicated that this change had limited impact due to interface issues and lack of uptake.

#### Education and training

Although many medical providers had worked in conflict settings before, participants questioned whether some of the staff deployed by NGOs had appropriate training or experience for an austere conflict setting like Mosul. Some felt that expatriate surgeons were undertaking time and resource-intensive definitive surgeries more appropriate for a stable, civilian setting rather than performing damage control surgery. In other cases, respondents said providers were performing unnecessary procedures that led to avoidable complications, such as wound infections and fistulas. However, data are not available to assess such statements. At both the TSP and hospital level, several organizations undertook medical training efforts with Iraqi physicians and nurses, although the quality and outcome of these trainings are largely unknown.

#### Research

In planning documents, organizers clearly acknowledged the importance of improving data quality and completeness so that it could be fed back into the pathway to optimize its functioning. In practice, however, data challenges limited such efforts. Some respondents felt that data collection would have benefited from greater input by medical providers with first-hand experience in battlefield medical care, as well as consultations with military and civilian trauma experts, to determine what type of data to collect, how to analyze the data, and how to use findings to improve the response. Several respondents also felt that earlier and larger investments should have been made in hiring monitoring and evaluation specialists to guide data collection and analyses that would have led to real-time enhancements in the pathway.

## Discussion

The Mosul civilian trauma response represented a novel effort by humanitarian actors to apply aspects of military battlefield trauma systems to improve access to care for severely injured civilians and avert an even greater humanitarian catastrophe in Mosul. This approach, implemented in real-time and under great pressure as gaps in trauma care became apparent, may have helped save up to 1500–1800 lives, according to a recent case study on the response [20, 21]. As a first-of-its-kind approach, it has attracted significant attention and debate within the humanitarian community and raised important questions about the extent to which trauma systems advances can be adapted by humanitarians in conflict settings.

We identified several areas where trauma systems concepts were effectively incorporated into the Mosul response. The organization of medical capacities into echelons of care, starting at the TSP level near the frontlines and continuing through field and referral hospitals, created a pathway that allowed civilians to receive care in a highly challenging, insecure environment where frontline services were otherwise lacking. The placement of TSPs near the frontlines clearly pushed care closer to the point of injury, as most civilian casualties were being evacuated by military vehicles and would not have had the means to reach care farther away. From a clinical perspective, efforts were made to define appropriate activities at each level of care; for example, WHO developed TSP guidelines for evidence-based pre-hospital care interventions, such as tourniquet placement and fluid resuscitation. Coordination among actors was encouraged and supported through a variety of mechanisms, including trauma working group meetings as well as intelligence and logistical support from the UN system.

However, the study found that important components that link echelons of care and have underpinned achievements of military battlefield trauma systems were difficult to implement in the Mosul context [11–13]. En-route medical care was limited by a lack of stocked ambulances and trained medical personnel, meaning that some patients likely suffered disruptions in care during transport, and air evacuation was not available. Capacity for post-operative care and rehabilitation was scarce, leading to patients being discharged without follow-up or rehabilitative care. Data collection was impacted by inconsistent reporting and lack of patient tracking, limiting conclusions about the response’s overall effectiveness. Field coordination was under-resourced, often relying upon a single individual to make decisions about where to send patients. Understandably, these challenges must be viewed within the context of planners needing to adapt quickly in a highly insecure environment and attempting such an ambitious response for the first time. But identifying such gaps may inform and help strengthen future responses.

Several limitations of this study should be acknowledged. The study was retrospective, as the authors did not directly observe the trauma response in real-time. Although efforts were made to interview as many direct participants as possible, some viewpoints may have been missed. Because interviews were limited to the organizations participating in the UN response, important perspectives, including those of Iraqi beneficiaries, local health authorities and providers, and other NGOs providing medical care during the response, were not included. Interview responses may have been affected by recall bias. The framework used to guide this study, although offering a systematic approach for conceptualizing trauma systems, has its own limitations, including being agnostic on the logistical and ethical complexities of implementation in other contexts. Finally, this analysis does not include quantitative data. Although quantitative data were collected by WHO and implementing partners, these data have limitations, discussed elsewhere [24, 25] and were excluded for this analysis. The lack of patient tracking in particular limits conclusions on continuity of care and patient outcomes.

The applicability of the Mosul approach to future conflicts is now being widely debated. In the Mosul response, donor interest, resource availability, and strong intelligence and security support from parties to the conflict (e.g. U.S. and European countries) were important, even essential, enabling factors. Whether these resources will be present in other humanitarian responses is an open question – and may well not be when high-income countries are less invested. Moreover, the recent achievements of military trauma systems in reducing battlefield mortality have drawn upon many technological advances, including the use of blood products in far forward positions, the reliance on airpower for rapid evacuation of casualties, and the development of sophisticated trauma registries that have allowed for the identification of suboptimal care and real-time improvements. A recent analysis by Howell et al. (2019), reviewing U.S. servicemember casualties in Iraq and Afghanistan, found that improvements in blood product availability, tourniquet use, and reduction in pre-hospital transport times accounted for nearly half of the reduction in case fatality in those conflicts [5]. Some observers have raised concerns that, in the absence of such advances, echeloning care may be counterproductive or even harmful if such echelons delay rather than expedite access to appropriate care.

Nonetheless, many humanitarian groups are now exploring ways to bring trauma care closer to the point of injury and improve civilian access to treatment. In recent years, MSF and ICRC have invested in mobile surgical units in conflict settings, and referrals from NGO-run field hospitals to facilities offering a higher level of care have been documented in various contexts [9, 10, 14, 15]. Given this interest, there is clearly a need to better understand how advances from military trauma systems can be adapted by humanitarians given the resource limitations and logistical challenges they face. A consensus framework for humanitarian responses for conflict was recently published which advances this agenda even further with greater detail [23].

There is also the contentious question of who should provide such care. During the Mosul response, many humanitarian organizations raised concerns that frontline trauma care is and should remain the responsibility of the warring parties under the Geneva Conventions and its Protocols, and that the willingness of the UN and humanitarian NGOs to “step in” and fill this void created a worrisome precedent, such that militaries may feel more comfortable outsourcing their responsibilities to humanitarians in future conflicts. These concerns deserve further consideration but are outside the scope of this article. Nonetheless, it is highly likely that in future conflicts, NGOs will continue to face questions about how to apply insights from military trauma systems to their actions, and what to do when professional militaries cannot, or will not, provide such care.

Although every conflict is unique and requires a contextually appropriate response, some generalizable opportunities may already exist. Guidelines could be developed to identify evidence-based interventions at different echelons, and specify the resources needed to support them. Opportunities for improving the availability of blood transfusions for civilians could be explored. Field coordination could be improved through basic investments in communication technology and software. En-route care could be strengthened by examining existing global procurement options for ambulances, supporting early assessments of transportation infrastructure, and funding basic training programs for local paramedics, as the ICRC has done in many previous conflicts. Data collection could be improved by identifying appropriate indicators and methodologies in advance, supporting the hiring of monitoring and evaluation specialists, and by making modest investments in patient tracking systems, modelled after the UK or US trauma registries and establishing guidelines for data ownership and access in advance. Many of these efforts would have the greatest impact if they begin now, before another international emergency trauma response is required. A good starting point would be to convene humanitarian, civilian, and trauma experts to discuss these points, develop guidelines, and endorse a research agenda for the future.

## Conclusions

The Mosul trauma pathway evolved to address critical gaps in trauma care during the Battle of Mosul. It adapted the concept of echelons of care from western military practice to push humanitarians closer to the frontlines and improve access to care for injured civilians. Although efforts were made to incorporate some of the integrative components (e.g. evidence-based pre-hospital care, transportation, and data collection) that have enabled recent achievements by military trauma systems, many of these proved difficult for humanitarians to implement in the Mosul context. Further discussion and research are needed to determine how trauma systems insights can be adapted in future humanitarian responses given resource, logistical, and security constraints, as well as to clarify the responsibilities of various actors.

## Supplementary information

**Additional file 1.** List Interviews.

**Additional file 2.** Semi-structured Interview Questionnaire for Participating Organizations.

**Additional file 3.** List of Documents Reviewed.

## Data Availability

The datasets generated and/or analysed during the current study are not publicly available due the conditions under which qualitative interviews were conducted for this study.
