# Supplementary material for: Applying trauma systems concepts to humanitarian battlefield care: a qualitative analysis of the Mosul trauma pathway
Source: Confl Health. 2020 Feb 4;14:5. doi: 10.1186/s13031-019-0249-2 (PMC7001520; doi:10.1186/s13031-019-0249-2)
Supplement: Supplementary file 1 — Additional file 1. List Interviews. [file 13031_2019_249_MOESM1_ESM.docx]

Additional file 1. List Interviews

| List of Interviews By Organization or Affiliation (# interviewed) |
| --- |
| Aspen Medical (3) |
| CADUS (1) |
| Emergency Hospital, Erbil (1) |
| European Civil Protection and Humanitarian Aid Operations (ECHO) (2) |
| Free Burma Rangers (1) |
| Global Response Management (2) |
| Handicap International (2) |
| Human Rights Watch (2) |
| International Committee of the Red Cross (ICRC): Geneva and Irbil (4) |
| International Federation of Red Cross and Red Crescent Societies (IFRC) (1) |
| IOM: Geneva and Erbil (2) |
| Karolinska Institute, Sweden (1) |
| Médecins Sans Frontières- Belgium/OCB : Brussels and Irbil (3) |
| Médecins Sans Frontières -Swiss/OCG: Geneva and Irbil (3) |
| Ninewah DOH (1) |
| NYC Medics (3) |
| Samaritan’s Purse (4) |
| U.S. Military/Coalition Partners (4) |
| UNFPA, Iraq (1) |
| UNHCR, Iraq (1) |
| United Nations Humanitarian Coordinator, Iraq (1) |
| UN-OCHA CivMil (2) |
| USAID/OFDA: DART Team, Iraq; Washington D.C. (3) |
| World Health Organization, Geneva and Irbil (6) |
