# Supplementary material for: Applying trauma systems concepts to humanitarian battlefield care: a qualitative analysis of the Mosul trauma pathway
Source: Confl Health. 2020 Feb 4;14:5. doi: 10.1186/s13031-019-0249-2 (PMC7001520; doi:10.1186/s13031-019-0249-2)
Supplement: Supplementary file 2 — Additional file 2. Semi-structured Interview Questionnaire for Participating Organizations. [file 13031_2019_249_MOESM2_ESM.docx]

Additional file 2. Semi-structured Interview Questionnaire for Participating Organizations

| Theme | Questions |
| --- | --- |
| **Decision to Participate and Planning** | - How did your involvement with the response begin? How did you learn about it? - Why did you decide to participate? What principles or considerations guided your decision? - Did you have reservations about participating? If so, what were those reservations? How were they addressed/answered? - What prior experience, if any, did your organization have in providing medical care during active conflict? - What, if any, background research did you do prior to deployment to better understand the context in which you would be working? - Prior to deployment, what were the main challenges you anticipated working in or around Mosul? - Which type of setting (TSP, field hospital, etc.) were you deployed to? How did you decide to provide this type of care/activity? - When did your activities begin (and end) at that site? - How long did it take to set up your site after arrival? |
| **Security and Logistics** | - What security assessments did you or your organization do before deploying? - How would you describe the security arrangement your site? - What security precautions were in place? What physical or protective barriers did you have in place? - Who provided security? - What communication did you have with other actors (military, UN, fellow NGOs, etc.) to obtain information regarding military activities or security threats? - Did security threats or incidents ever disrupt your activities? If so, how? - Was anyone at your site/on your team ever injured? |
| **Staffing** | - Describe the composition of your medical team. How many personnel did you have? In what roles? - Did your organization have a process for assessing provider qualifications? Were there specific experience requirements? - Were you (and others) required to undergo any training courses (ACLS, conflict medicine, etc.) prior to deployment? - Were participants paid? If so, what type of salary or stipend did you receive? Where did payments come from? - Do you feel that the skills and experience of participants were appropriate for the context? - At any point did you feel like your organization lacked the technical capacity, personnel, or resources to provide adequate care? - Do you believe your team fulfilled its intended objectives? |
| **Resources and supplies** | - Did you feel like your site had adequate equipment and supplies? If no, what types of equipment or supplies were needed that were not available? - Did you ever experience shortages—if so, what supplies, and for how long? - What system was in place for ensuring an adequate supply chain? What was the process for requesting additional resources? How well did this system/process work? How could it have been improved? - If relevant, did your site have electricity and water? Did you require connections to the electricity grid or running water? Did you ever experience disruptions in electricity or water that affected your availability to provide services? - How adaptable was your site to changing situations? Describe a situation in which your site/group had to adapt quickly and how it managed that situation. - Did you feel like your site was adequately staffed? If no, what additional personnel (type, capacity) were needed—and why? If you did have a need for additional personnel, was there a process for requesting extra staff, and how quickly were those requests met? |
| **Management and patient care** | - Describe your protocol for triaging patients upon arrival at your site(s). How did you develop this protocol? How did you teach this protocol to your volunteers/participants? - Were there any areas in your triage protocol that needed improvement/strengthening? - What was your approach to managing civilians, Iraqi/Kurdish fighters, and ISIS fighters? Did you make any distinction between these groups in terms of your treatment or management? - Describe the medical activities provided at your site. How did you decide to provide this specific set of activities? - Did your team follow guidelines or protocols for patient care? If so, what were these guidelines? How was the decision made to use those guidelines specifically? - Do you believe your team followed accepted standards and guidelines for patient care at all times? If not, what standards or guidelines could be been strengthened? - Describe the process for discharging patients from your care. Where did patients go? Did opportunities for follow-up exist? - For patients with complex injuries requiring further operations or rehabilitation, what services were available? |
| **Communication and Coordination** | - If a decision was made that a patient needed a higher level of care, how did you refer them up the chain of care? Who did you contact? How did you contact them? - Describe the agility of the chain—did TSPs always refer to the same field hospital, or were there multiple options depending upon conditions and capacity? - Likewise, did field hospitals always refer to the same tertiary center? - How would you describe communication between TSPs, field hospitals, and tertiary referral centers? Were you able to contact other providers in real-time to determine appropriateness of transfer? - Once a decision was made to send the patient to the next level of care, how quick was the referral process? Were than any major sources of delays (e.g. ambulance availability, capacity of receiving hospital, etc.) or bottlenecks? - Did you feel like ambulances were readily available? Was there always an ambulance present on site to transfer patients? |
| **Data Collection** | - What type of data did you collect at your site(s)? - How did you decide which categories of data to collect? - Did you collect data on the number and type of cases (ex lap, c-section, amputation, etc.) performed? - Did you collect any data on outcomes or complications? - Did you collect data on where patients went following their discharge from your site? - How did you collect data (paper, electronic)? Who was responsible for data collection at your sites? - To whom did you report your data? How frequently did your report data (daily, weekly)? - Do you feel that reported data was used to inform the trauma response and make appropriate and timely changes when needed? - What challenges with data collection and reporting, if any, did you experience in the field? |
| **Sustainability and Capacity Building** | - To what extent did your organization engage in training of local Iraqi or Kurdish health workers? Describe the extent of your training programs. How did you prioritize the type of training provided? - To what extent did your organization’s teams incorporate local health workers? In what capacity did these local health workers serve? - How would you characterize the cooperation of foreign and local health workers? Were there any issues or challenges that needed to be addressed or were difficult to overcome? - Did you develop plans to transition operation of your sites to Iraqi counterparts upon cessation of your activities? If yes, describe these plans. To what extent have these plans been executed? |
