# Supplementary material for: Applying trauma systems concepts to humanitarian battlefield care: a qualitative analysis of the Mosul trauma pathway
Source: Confl Health. 2020 Feb 4;14:5. doi: 10.1186/s13031-019-0249-2 (PMC7001520; doi:10.1186/s13031-019-0249-2)
Supplement: Supplementary file 3 — Additional file 3. List of Documents Reviewed. [file 13031_2019_249_MOESM3_ESM.docx]

Additional file 3. List of Documents Reviewed

1. News Articles

Arango, T. and Cooper, H. U.S. Investigating Mosul Strikes Said to Have Killed Up to 200 Civilians. New York Times, March 24, 2017. <https://www.nytimes.com/2017/03/24/world/middleeast/us-iraq-mosul-investigation-airstrike-civilian-deaths.html?_r=1>

Astor, M. Mosul operation to test UN humanitarian efforts. Associated Press, October 17, 2017. <https://apnews.com/192a7029ad9c498c87eecdd083e0161a>.

Babb, C. Americans Advancing on Mosul with Iraqis and Kurds. Voice of America. October 18, 2016. <https://www.voanews.com/a/iraqi-kurdish-fighters-make-gains-in-battle-to-retake-mosul/3556203.html>

Behn, S. 6,500 Coalition Troops in Iraq; U.S. Wants More. Voice of America, February 2, 2016. <https://www.voanews.com/a/sixty-five-hundred-coalition-troops-in-iraq-us-wants-more/3172721.html>

Chulov, M, et al. Battle for Mosul: Iraqi forces converge in decisive battle against ISIL. The Guardian, October 17, 2016. <https://www.theguardian.com/world/2016/oct/17/kurds-lead-advance-on-mosul-in-decisive-battle-for-iraqs-second-city>

Chulov, M. and Hawramy, F. Battle for Mosul: Iraqi forces converge in decisive battle against ISIL. The Guardian, October 18, 2017. <https://www.theguardian.com/world/2016/oct/17/kurds-lead-advance-on-mosul-in-decisive-battle-for-iraqs-second-city>

Fordham, Alice. No One Was Prepared To Care for So Many Wounded In Mosul. NPR. March 5, 2017. <http://www.npr.org/sections/goatsandsoda/2017/03/05/518360464/no-one-was-prepared-to-care-for-so-many-wounded-in-mosul>

George, S. In final stages of Mosul battle, U.S. troops take on Expanded Role. Associated Press, July 1, 2017. <http://www.chicagotribune.com/news/nationworld/ct-islamic-state-mosul-20170701-story.html>

George, S. Mosul is a graveyard: Final IS battle kills 9,000 civilians. Dec. 21, 2017. [https://www.apnews.com/ bbea7094fb954838a2](https://www.apnews.com/%20bbea7094fb954838a2)fdc11278d65460

George, S. and B. Szlanko. Iraqi forces prepare for assault. The Associated Press. August 13, 2016. <http://www.businessinsider.com/ap-un-100000-displaced-as-iraqi-forces-poise-for-mosul-battle-2016-8>

Gibbons-Neff, T. U.S. failed to keep proper track of more than $1 billion in weapons and equipment in Iraq. Washington Post, May 24, 2017. <https://www.washingtonpost.com/news/checkpoint/wp/2017/05/24/u-s-failed-to-keep-proper-track-of-more-than-1-billion-in-weapons-and-equipment-in-iraq/?utm_term=.a71449e977c5>

Graham-Harrison, E. Could the liberation of Mosul lead to a million fleeing to Iraqi Kurdistan? The Guardian, August 27, 2016. [https://www.theguardian.com/world/2016/aug/27/iraq-kurdistan-mosul-battle-refugee-crISIL](https://www.theguardian.com/world/2016/aug/27/iraq-kurdistan-mosul-battle-refugee-crisis)

Graham-Harrison, Emma. Could the liberation of Mosul to lead to one million fleeing to Iraqi Kurdistan? The Guardian, August 27, 2016. <https://www.theguardian.com/world/2016/aug/27/iraq-kurdistan-mosul-battle-refugee-crISIL>

Handicap International. Emergency Rehabilitation Response Mosul Crisis. Rapid Needs Assessment. Handicap International: Iraq. January 2017.

Hendawi, H. and Abdul-Zahra, Q. Retaking militant-held Iraqi city likely to cost. Associated Press, August 1, 2016. <http://www.gazettenet.com/Retaking-city-tricky-3775342>

Meek, J, Newmani, H, Ross, B, Schwartz, R. US ignores evidence of atrocities by blacklisted Iraqi military unit. ABC News, May 31, 2017. <http://abcnews.go.com/International/us-ignores-evidence-atrocities-blacklisted-iraqi-military-unit/story?id=47745913>

Michaels, J. Iraqi forces in Mosul see deadliest urban combat since World War II. USA Today, March 29, 2017. <https://www.usatoday.com/story/news/world/2017/03/29/united-states-mosul-ISIL-deadly-combat-world-war-ii/99787764/>

Sisk, R. More Than 100 US Troops Move Forward With Mosul Attack Force. Military.com, October 18, 2016. <http://www.military.com/daily-news/2016/10/18/100-us-troops-move-forward-mosul-attack-force.html>

Slemrod, A. The failure in Fallujah. IRIN, June 28, 2016. <https://www.irinnews.org/analysis/2016/06/28/failure-Fallujah>.

Smith, J. International Red Cross to drastically cut Afghan operations after attacks. Reuters, October 9, 2017. <https://www.reuters.com/article/us-afghanistan-icrc/international-red-cross-to-drastically-cut-afghan-operations-after-attacks-idUSKBN1CE0AG?il=0>

2. Operational Documents

Ammar, W. and Ryan, M. IOAC Iraq Mission Report. Iraq Oversight and Advisory Committee for WHO Health Emergencies Program. March 22-24, 2017.

Médecins Sans Frontières. Overview Projects in Mosul and Surrounding Areas. April 30, 2017. <http://www.ncciraq.org/en/ngos/ngo-activities/item/19516-msf-overview-projects-in-mosul-and-surrounding-areas>

Médecins Sans Frontières. Iraq: MSF expands its medical services in the conflicted city of Mosul. May 4, 2017. <http://www.msf.ca/en/article/iraq-msf-expands-its-medical-services-in-the-conflicted-city-of-mosul>

Médecins Sans Frontières. Rebuilding lives shattered by war: Testimonies from MSF's Hamdaniya hospital in Iraq. May 4, 2017. <http://www.msf.ca/en/article/rebuilding-lives-shattered-by-war-testimonies-from-msf-s-hamdaniya-hospital-in-iraq>

OCHA. Humanitarian Funding Update. September 2016. <https://reliefweb.int/report/world/humanitarian-funding-update-september-2016-united-nations-coordinated-appeals>

OCHA. What are Humanitarian Principles? June 2012. <https://docs.unocha.org/sites/dms/.../OOM-humanitarianprinciples_eng_June12.pdf>

UN Habitat. City Profile of Mosul, Iraq: Multi-sector Assessment of a City Under Siege. October 2016. <https://reliefweb.int/sites/reliefweb.int/files/resources/UN-Habitat_MosulCityProfile_V5.pdf>

UNAMI. Press Briefing, Lise Grande. July 17, 2017. <http://webtv.un.org/watch/lise-grande-unami-on-the-situation-in-iraq-press-conference-17-july-2017/5510054178001/?term>=

United Nations. After Mosul victory, senior UN officials detail Iraq’s political and humanitarian needs. July 17, 2017. <http://www.un.org/apps/news/story.asp?NewsID=57192#.WfY807pFw2w>

United Nations. Joint Statement on One Month of Military Operations to Retake Mosul. November 17, 2016.

United Nations. Mosul Flash Appeal. July 20, 2016. <https://reliefweb.int/sites/reliefweb.int/files/resources/mosul_flash_appeal_final_web.pdf>

US Defense Department Briefing. September 8, 2016. <https://www.defense.gov/News/Transcripts/Transcript-View/Article/938183/department-of-defense-press-briefing-by-col-dorrian-via-teleconference-from-bag/>

USAID. Iraq: Complex Emergency. Fact Sheet #2: Fiscal Year 2017. Mar h 31, 2017.

USAID. Iraq: Complex Emergency. Fact Sheet #3: Fiscal Year 2016. April 11, 2016.

USAID. Iraq: Complex Emergency. Fact Sheet #3: Fiscal Year 2017. May 5, 2017.

USAID. Iraq: Complex Emergency. Fact Sheet #4: Fiscal Year 2017. June 9, 2017.

USAID. Iraq: Complex Emergency. Fact Sheet #5: Fiscal Year 2016. September 14, 2016.

USAID. Iraq: Complex Emergency. Fact Sheet #5: Fiscal Year 2017. July 14, 2017.

USAID. Iraq: Complex Emergency. Fact Sheet #6: Fiscal Year 2016. September 30, 2016.

USAID. Iraq: Complex Emergency. Fact Sheet #6: Fiscal Year 2017. August 11, 2017.

WHO. As Trauma Needs Escalate in West Mosul, More Ambulances Are Deployed. April 24, 2017.

WHO. Attacks on healthcare on the rise in Afghanistan. <http://www.emro.who.int/afg/afghanistan-news/attacks-on-healthcare-on-the-rise-in-afghanistan.html>

WHO. Attacks on Healthcare: Dashboard. 2016. <http://www.who.int/emergencies/attacks-on-health-care/attacks_dashboard_2016_updated-June2017.pdf?ua=1>

WHO. Briefing Notes. December 2, 2016.

WHO. Civilians Caught in the Crossfire: WHO Supports Trauma Care Services for People in and around Mosul. November 9, 2016.

WHO. Delivering Healthcare in Newly Liberated Areas: WHO Mobile Medical Clinics Reach People in Need. November 13, 2016.

WHO. Efforts Ongoing to Provide Trauma Care to People in Need in Mosul, Iraq. January 25, 2017.

WHO. Fifteen Ambulances Airlifted into Iraq to Serve Trauma Needs in Mosul. March 20, 2017.

WHO. Global Health Cluster Partner Meeting: Note for the Record. April 7-8, 2017. <http://www.who.int/health-cluster/about/structure/GHC-Partner-Meeting-Apr2017-NFR.pdf?ua=1>

WHO. Health Attacks Dashboard: 2016. <http://www.who.int/emergencies/attacks-on-health-care/attacks_dashboard_2016_updated-June2017.pdf?ua=1>

WHO. Iraq Humanitarian Crisis Presentation. March 2017. http://www.who.int/health-cluster/about/structure/Iraq2-web.pdf?ua=1

WHO. Mosul City Expected Displacement and Health Preparedness. Health Cluster Iraq. October 17, 2016.

WHO. Mosul Operation: Casualty Cases to Emergency and West Emergency Hospitals, Erbil Iraq (17 October to 17 December 2016). Infographic, Version 12.

WHO. Situation Report 11: Iraq Crisis. November 2016.

WHO. Special Situation Report, Mosul Crisis, Iraq. Issue 1. January 1-7, 2017.

WHO. Special Situation Report, Mosul Crisis, Iraq. Issue 2. January 7-14, 2017.

WHO. Special Situation Report, Mosul Crisis, Iraq. Issue 25. July 9-23, 2017.

WHO. Special Situation Report, Mosul Crisis, Iraq. Issue 25. June 25-July 8, 2017.

WHO. Special Situation Report, Mosul Crisis, Iraq. Issue 3. January 15-21, 2017.

WHO. Trauma Care Now Available in Bartella. January 23, 2017.

WHO. Trauma care plan near frontlines during Mosul Offensive: Concept of operations planning paper (draft) as of 25/10/16.

WHO. Trauma care plan near frontlines during Mosul Offensive: Updated Concept of operations planning as of 23/11/16.

WHO. Trauma care and referral plan near frontlines during Mosul Offensive: Updated Concept of operations planning as of 18/03/17.

WHO. Trauma Field Hospital in Hammam Al-Alil goes 24/7. April 27, 2017.

WHO. Trauma Working group minutes. April 19, 2017.

WHO. Trauma Working group minutes. April 26, 2017.

WHO. Trauma Working group minutes. May 3, 2017.

WHO. Trauma Working group minutes. May 10, 2017.

WHO. Trauma Working group minutes. May 17, 2017.

WHO. Trauma Working group minutes. May 24, 2017.

WHO. Trauma Working group minutes. May 31, 2017.

WHO. Trauma Working group minutes. June 8, 2017.

WHO. Trauma Working group minutes. June 21, 2017.

WHO. Trauma Working group minutes. July 10, 2017.

WHO. WHO and Partners Gear up to Safeguard Lives of Displaced Persons Fleeing Mosul. October 26, 2016.

WHO. WHO Establishes Raid Response Teams to Safeguard Health of Newly Displaced People from Mosul, Iraq. Nov 6, 2016.

WHO. WHO Press Notes: UN Briefing. November 8, 2016.

WHO. WHO Press Notes: UN Briefing. October 25, 2016.

WHO. WHO Scales up Response to Critical Trauma Needs as Plans for West Mosul Operations Intensify. January 30, 2017.

WHO. WHO Scales Up Trauma Care Services for Injured People from Mosul, Iraq. November 23, 2016.

WHO. WHO Supports Iraqi Capacity on Mass Casualty Management of Chemical Agents. October 24, 2016.

WHO. WHO supports Kurdistan Ministry of Health by Training Doctors and Paramedics to Respond to Civilian Casualties in Mosul. December 23, 2016.

WHO. WHO’s Response to Trauma Cases Saves Hundreds of Lives in Iraq. January 5, 2017.

WHO. Within Hours of Opening Its Doors, Athbah Trauma Field Hospital Treats Casualties from West Mosul. March 28, 2017.

3. Academic and Peer-Review Literature

ACS (American College of Surgeons). 2008. *Regional trauma systems: Optimal elements, integration, and assessment, American College of Surgeons Committee on Trauma: Systems consultation guide*. Chicago, IL: ACS.

American Trauma Society, 2004. Trauma System Agenda for the Future. [https://www.ems.gov/pdf/advancing-ems-systems/Provider- Resources/EMS_Trauma_Agenda.pdf](https://www.ems.gov/pdf/advancing-ems-systems/Provider-%20Resources/EMS_Trauma_Agenda.pdf)

Arango, T. and Cooper, H. U.S. Investigating Mosul Strikes Said to Have Killed Up to 200 Civilians. New York Times, March 24,

Bailey, J., M. A. Spott, G. P. Costanzo, J. R. Dunne, W. Dorlac, and B. J. Eastridge. 2012a. *Joint Trauma System: Development, conceptual framework, and optimal elements.* San Antonio, TX: Fort Sam Houston, U.S. Department of Defense, U.S. Army Institute for Surgical Research

Berwick D, Downey A, Cornett E, eds. A National Trauma Care System: Integrating Military and Civilian Trauma Systems to Achieve Zero Preventable Deaths After Injury. Washington (DC): [National Academies Press (US)](http://www.nap.edu/); 2016. <https://www.ncbi.nlm.nih.gov/books/NBK390321/>

Blackbourne, L. H., D. G. Baer, B. J. Eastridge, F. K. Butler, J. C. Wenke, R. G. Hale, R. S. Kotwal, L. R. Brosch, V. S. Bebarta, M. M. Knudson, J. R. Ficke, D. Jenkins, and J. B. Holcomb. 2012. Military medical revolution: Military trauma system. *Journal of the American Academy of Orthopaedic Surgeons* 73(6 Suppl. 5):S388-S394.

[Blyth](https://www.ncbi.nlm.nih.gov/pubmed/?term=Blyth%20DM%5BAuthor%5D&cauthor=true&cauthor_uid=26406435), D., [Heather C. Yun](https://www.ncbi.nlm.nih.gov/pubmed/?term=Yun%20HC%5BAuthor%5D&cauthor=true&cauthor_uid=26406435), [David R. Tribble](https://www.ncbi.nlm.nih.gov/pubmed/?term=Tribble%20DR%5BAuthor%5D&cauthor=true&cauthor_uid=26406435), and [Clinton K. Murray](https://www.ncbi.nlm.nih.gov/pubmed/?term=Murray%20CK%5BAuthor%5D&cauthor=true&cauthor_uid=26406435). Lessons of War: Combat-related Injury Infections during the Vietnam War and Operation Iraqi and Enduring Freedom. [J Trauma Acute Care Surg](https://www.ncbi.nlm.nih.gov/pmc/articles/PMC4586048/). J Trauma Acute Care Surg. 2015 Oct; 79: S227–S235.

Butler FK, Blackbourne LH: Battlefield trauma care then and now: a decade of tactical combat casualty care. J Trauma Acute Care Surg 2012; 73: S395–S402.

Butler FK. Two Decades of Saving Lives on the Battlefield: Tactical Combat Casualty Care Turns 20. [Mil Med.](https://www.ncbi.nlm.nih.gov/pubmed/28290925) 2017 Mar;182(3):e1563-e1568.

Butler, F. K., D. J. Smith, and R. H. Carmona. 2015. Implementing and preserving the advances in combat casualty care from Iraq and Afghanistan throughout the US military. *Journal of Trauma and Acute Care Surgery* 79(2):321-326.

Childers R and Parker, P. In a stable battlefield, avoid using austere surgical units to meet the golden hour of trauma time to care goal.[Injury.](https://www.ncbi.nlm.nih.gov/pubmed/?term=childers+and+golden+hour) 2017 Nov;48(11):2379-2382.

[Chu K](https://www.ncbi.nlm.nih.gov/pubmed/?term=Chu%20K%5BAuthor%5D&cauthor=true&cauthor_uid=20398250), [Havet P](https://www.ncbi.nlm.nih.gov/pubmed/?term=Havet%20P%5BAuthor%5D&cauthor=true&cauthor_uid=20398250), [Ford N](https://www.ncbi.nlm.nih.gov/pubmed/?term=Ford%20N%5BAuthor%5D&cauthor=true&cauthor_uid=20398250), [Trelles M](https://www.ncbi.nlm.nih.gov/pubmed/?term=Trelles%20M%5BAuthor%5D&cauthor=true&cauthor_uid=20398250). Surgical care for the direct and indirect victims of violence in the eastern Democratic Republic of Congo. [Confl Health.](https://www.ncbi.nlm.nih.gov/pubmed/20398250) 2010 Apr 14;4:6. doi: 10.1186/1752-1505-4-6.

[Chu K](https://www.ncbi.nlm.nih.gov/pubmed/?term=Chu%20K%5BAuthor%5D&cauthor=true&cauthor_uid=19672649), [Rosseel P](https://www.ncbi.nlm.nih.gov/pubmed/?term=Rosseel%20P%5BAuthor%5D&cauthor=true&cauthor_uid=19672649), [Trelles M](https://www.ncbi.nlm.nih.gov/pubmed/?term=Trelles%20M%5BAuthor%5D&cauthor=true&cauthor_uid=19672649), [Gielis P](https://www.ncbi.nlm.nih.gov/pubmed/?term=Gielis%20P%5BAuthor%5D&cauthor=true&cauthor_uid=19672649).Surgeons without borders: a brief history of surgery at Médecins Sans Frontières. [J Surg.](https://www.ncbi.nlm.nih.gov/pubmed/19672649) 2010 Mar;34(3):411-4.

Coupland, R. and Howell, P. An experience of war surgery and wounds presenting after 3 days on the border of Afghanistan. lnjury(1988) 19,259-262.

[Cross KP](https://www-ncbi-nlm-nih-gov.ezp.welch.jhmi.edu/pubmed/?term=Cross%20KP%5BAuthor%5D&cauthor=true&cauthor_uid=25153986), [Petry MJ](https://www-ncbi-nlm-nih-gov.ezp.welch.jhmi.edu/pubmed/?term=Petry%20MJ%5BAuthor%5D&cauthor=true&cauthor_uid=25153986), [Cicero MX](https://www-ncbi-nlm-nih-gov.ezp.welch.jhmi.edu/pubmed/?term=Cicero%20MX%5BAuthor%5D&cauthor=true&cauthor_uid=25153986). A better START for low-acuity victims: data-driven refinement of mass casualty triage. [Prehosp Emerg Care.](https://www-ncbi-nlm-nih-gov.ezp.welch.jhmi.edu/pubmed/?term=A+Better+START+for+Low-acuity+Victims%3A+Data-driven+Refinement+of+Mass+Casualty+Triage) 2015 Apr-Jun;19(2):272-8.

Eastridge, B. J., D. Jenkins, S. Flaherty, H. Schiller, and J. B. Holcomb. 2006. Trauma system development in a theater of war: Experiences from Operation Iraqi Freedom and Operation Enduring Freedom. *Journal of Trauma* 61(6):1366-1372.

Eastridge, B. J., G. Costanzo, D. Jenkins, M. A. Spott, C. Wade, D. Greydanus, S. Flaherty, J. Rappold, J. Dunne, J. B. Holcomb, and L. H. Blackbourne. 2009. Impact of joint theater trauma system initiatives on battlefield injury outcomes. *American Journal of Surgery* 198(6):852-857.

Eastridge, B. J., R. L. Mabry, P. Seguin, J. Cantrell, T. Tops, P. Uribe, O. Mallett, T. Zubko, L. Oetjen-Gerdes, T. E. Rasmussen, F. K. Butler, R. S. Kotwal, J. B. Holcomb, C. Wade, H. Champion, M. Lawnick, L. Moores, and L. H. Blackbourne. 2012. Death on the battlefield (2001-2011): Implications for the future of combat casualty care. *Journal of Trauma and Acute Care Surgery* 73(6 Suppl. 5):S431-S437.

[Edwards MJ](https://www.ncbi.nlm.nih.gov/pubmed/?term=Edwards%20MJ%5BAuthor%5D&cauthor=true&cauthor_uid=24745567), [Lustik M](https://www.ncbi.nlm.nih.gov/pubmed/?term=Lustik%20M%5BAuthor%5D&cauthor=true&cauthor_uid=24745567), [Burnett MW](https://www.ncbi.nlm.nih.gov/pubmed/?term=Burnett%20MW%5BAuthor%5D&cauthor=true&cauthor_uid=24745567), [Eichelberger M](https://www.ncbi.nlm.nih.gov/pubmed/?term=Eichelberger%20M%5BAuthor%5D&cauthor=true&cauthor_uid=24745567). Pediatric inpatient humanitarian care in combat: Iraq and Afghanistan 2002 to 2012. [J Am Coll Surg.](https://www.ncbi.nlm.nih.gov/pubmed/?term=mary+edwards+pediatric+inpatient+humanitarian+care+in+combat) 2014 May;218(5):1018-23.

Footer, K and Rubenstein, L. A Human Rights Approach to Health Care in Conflict. International Review of the Red Cross, 2013, 95(899): 1-21.

Gross, M. (2017) Saving Life, Limb, and Eyesight: Assessing the Medical Rules of Eligibility During Armed Conflict, The American Journal of Bioethics, 17:10, 40-52.

Holcomb JB, McMullen NR, Pearse L, et al: Causes of death in U.S. Special Operations forces in the global war on terror. Ann Surg 2007; 245: 986–91.

Holcomb JB. Major scientific lessons learned in the trauma field over the last two decades. [PLoS Med.](https://www.ncbi.nlm.nih.gov/pubmed/?term=holcomb+major+scientific+lessons+trauma) 2017 Jul 5;14(7):e1002339. doi: 10.1371/journal.pmed.1002339.

Kahn, C et al. Does START Triage Work? An Outcomes Assessment After a Disaster. [Annals of Emergency Medicine](http://www.sciencedirect.com.ezp.welch.jhmi.edu/science/journal/01960644). [Volume 54, Issue 3](http://www.sciencedirect.com.ezp.welch.jhmi.edu/science/journal/01960644/54/3), September 2009, Pages 424-430.e1

Korver, A. Outcome of war injured patients treated at first aid posts of the ICRC. Injury (1994) 25, (I), 25-30.

Kotwal, R. S., H. R. Montgomery, B. M. Kotwal, H. R. Champion, F. K. Butler Jr., R. L. Mabry, J. S. Cain, L. H. Blackbourne, K. K. Mechler, and J. B. Holcomb. 2011. Eliminating preventable death on the battlefield. *Archives of Surgery* 146(12):1350-1358.

Kotwal, R. S., J. T. Howard, J. A. Orman, B. W. Tarpey, J. A. Bailey, H. R. Champion, R. L. Mabry, J. B. Holcomb, and K. R. Gross. 2016. The effect of a golden hour policy on the morbidity and mortality of combat casualties. *JAMA Surgery* 151(1):15-24.

Kragh JF, Walters TJ, Baer DJ, et al: Survival with emergency tourniquet use to stop bleeding in major limb trauma. Ann Surg 2009; 249: 1–7.

Mann, N. C., R. J. Mullins, E. J. MacKenzie, G. J. Jurkovich, and C. N. Mock. 1999. Systematic review of published evidence regarding trauma system effectiveness. *Journal of Trauma* 47(Suppl. 3):S25-S33.

Maughon JS. An inquiry into the nature of wounds resulting in killed in action in Vietnam. Mil Med 1970; 135:8-13.

Moore, Lynne, Lavoie, André, Bourgeois, Gilles, and Lapointe, Jean. Journal of Trauma and Acute Care Surgery. Donabedian’s structure-process-outcome quality of care model: Validation in an integrated trauma system. Issue: Volume 78(6), June 2015, p 1168–1175.

[Morrison JJ](https://www-ncbi-nlm-nih-gov.ezp.welch.jhmi.edu/pubmed/?term=Morrison%20JJ%5BAuthor%5D&cauthor=true&cauthor_uid=23291661), [Oh J](https://www-ncbi-nlm-nih-gov.ezp.welch.jhmi.edu/pubmed/?term=Oh%20J%5BAuthor%5D&cauthor=true&cauthor_uid=23291661), [DuBose JJ](https://www-ncbi-nlm-nih-gov.ezp.welch.jhmi.edu/pubmed/?term=DuBose%20JJ%5BAuthor%5D&cauthor=true&cauthor_uid=23291661), [O'Reilly DJ](https://www-ncbi-nlm-nih-gov.ezp.welch.jhmi.edu/pubmed/?term=O'Reilly%20DJ%5BAuthor%5D&cauthor=true&cauthor_uid=23291661), [Russell RJ](https://www-ncbi-nlm-nih-gov.ezp.welch.jhmi.edu/pubmed/?term=Russell%20RJ%5BAuthor%5D&cauthor=true&cauthor_uid=23291661), [Blackbourne LH](https://www-ncbi-nlm-nih-gov.ezp.welch.jhmi.edu/pubmed/?term=Blackbourne%20LH%5BAuthor%5D&cauthor=true&cauthor_uid=23291661), [Midwinter MJ](https://www-ncbi-nlm-nih-gov.ezp.welch.jhmi.edu/pubmed/?term=Midwinter%20MJ%5BAuthor%5D&cauthor=true&cauthor_uid=23291661), [Rasmussen TE](https://www-ncbi-nlm-nih-gov.ezp.welch.jhmi.edu/pubmed/?term=Rasmussen%20TE%5BAuthor%5D&cauthor=true&cauthor_uid=23291661). En-route care capability from point of injury impacts mortality after severe wartime injury. [Ann Surg.](https://www-ncbi-nlm-nih-gov.ezp.welch.jhmi.edu/pubmed/23291661) 2013 Feb;257(2):330-4.

[Porta CR](https://www.ncbi.nlm.nih.gov/pubmed/?term=Porta%20CR%5BAuthor%5D&cauthor=true&cauthor_uid=24791642), [Robins R](https://www.ncbi.nlm.nih.gov/pubmed/?term=Robins%20R%5BAuthor%5D&cauthor=true&cauthor_uid=24791642), [Eastridge B](https://www.ncbi.nlm.nih.gov/pubmed/?term=Eastridge%20B%5BAuthor%5D&cauthor=true&cauthor_uid=24791642), [Holcomb J](https://www.ncbi.nlm.nih.gov/pubmed/?term=Holcomb%20J%5BAuthor%5D&cauthor=true&cauthor_uid=24791642), [Schreiber M](https://www.ncbi.nlm.nih.gov/pubmed/?term=Schreiber%20M%5BAuthor%5D&cauthor=true&cauthor_uid=24791642), [Martin M](https://www.ncbi.nlm.nih.gov/pubmed/?term=Martin%20M%5BAuthor%5D&cauthor=true&cauthor_uid=24791642). The hidden war: humanitarian surgery in a combat zone. [Am J Surg.](https://www.ncbi.nlm.nih.gov/pubmed/?term=porta+the+hidden+war) 2014 May;207(5):766-72.

[Rasmussen TE](https://www.ncbi.nlm.nih.gov/pubmed/?term=Rasmussen%20TE%5BAuthor%5D&cauthor=true&cauthor_uid=26406437), [Baer DG](https://www.ncbi.nlm.nih.gov/pubmed/?term=Baer%20DG%5BAuthor%5D&cauthor=true&cauthor_uid=26406437), [Cap AP](https://www.ncbi.nlm.nih.gov/pubmed/?term=Cap%20AP%5BAuthor%5D&cauthor=true&cauthor_uid=26406437), [Lein BC](https://www.ncbi.nlm.nih.gov/pubmed/?term=Lein%20BC%5BAuthor%5D&cauthor=true&cauthor_uid=26406437). Ahead of the curve: Sustained innovation for future combat casualty care. [J Trauma Acute Care Surg.](https://www.ncbi.nlm.nih.gov/pubmed/26406437) 2015 Oct;79(4 Suppl 2):S61-4.

Remick, K. and Elster, E. (2017). Trauma Care in Support of Global Military Operations. Joint Force Quarterly, July 2017.

Safeguarding Health in Conflict Coalition. Impunity Must End: Attacks on Health in 23 Countries in 2016. <https://www.safeguardinghealth.org/sites/shcc/files/SHCC2017final.pdf>

Sharon R. Weeks, John S. Oh, Eric A. Elster, Peter A. Learn. Humanitarian Surgical Care in the US Military Treatment Facilities in Afghanistan From 2002 to 2013. JAMA Surg. Published online September 13, 2017. doi:10.1001/jamasurg.2017.3142

Tien, H, et al. Advances in damage control resuscitation and surgery: implications on the organization of future military field forces. [Can J Surg](https://www.ncbi.nlm.nih.gov/pmc/articles/PMC4467505/). 2015 Jun; 58(3 Suppl 3): S91–S97.

Trelles M, Dominguez L, Tayler-Smith K, Kisswani K, Zerboni A, Vandenborre T, Dallatomasina S, Rahmoun A, Ferir MC. [Providing surgery in a war-torn context: the Médecins Sans Frontières experience in Syria.](https://www.ncbi.nlm.nih.gov/pubmed/26674297) Confl Health. 2015 Dec 15;9:36

[Valles P](https://www-ncbi-nlm-nih-gov.ezp.welch.jhmi.edu/pubmed/?term=Valles%20P%5BAuthor%5D&cauthor=true&cauthor_uid=27810881), [Van den Bergh R](https://www-ncbi-nlm-nih-gov.ezp.welch.jhmi.edu/pubmed/?term=Van%20den%20Bergh%20R%5BAuthor%5D&cauthor=true&cauthor_uid=27810881), [van den Boogaard W](https://www-ncbi-nlm-nih-gov.ezp.welch.jhmi.edu/pubmed/?term=van%20den%20Boogaard%20W%5BAuthor%5D&cauthor=true&cauthor_uid=27810881), [Tayler-Smith K](https://www-ncbi-nlm-nih-gov.ezp.welch.jhmi.edu/pubmed/?term=Tayler-Smith%20K%5BAuthor%5D&cauthor=true&cauthor_uid=27810881), [Gayraud O](https://www-ncbi-nlm-nih-gov.ezp.welch.jhmi.edu/pubmed/?term=Gayraud%20O%5BAuthor%5D&cauthor=true&cauthor_uid=27810881), [Mammozai BA](https://www-ncbi-nlm-nih-gov.ezp.welch.jhmi.edu/pubmed/?term=Mammozai%20BA%5BAuthor%5D&cauthor=true&cauthor_uid=27810881), [Nasim M](https://www-ncbi-nlm-nih-gov.ezp.welch.jhmi.edu/pubmed/?term=Nasim%20M%5BAuthor%5D&cauthor=true&cauthor_uid=27810881), [Cheréstal S](https://www-ncbi-nlm-nih-gov.ezp.welch.jhmi.edu/pubmed/?term=Cher%C3%A9stal%20S%5BAuthor%5D&cauthor=true&cauthor_uid=27810881), [Majuste A](https://www-ncbi-nlm-nih-gov.ezp.welch.jhmi.edu/pubmed/?term=Majuste%20A%5BAuthor%5D&cauthor=true&cauthor_uid=27810881), [Charles JP](https://www-ncbi-nlm-nih-gov.ezp.welch.jhmi.edu/pubmed/?term=Charles%20JP%5BAuthor%5D&cauthor=true&cauthor_uid=27810881), [Trelles M](https://www-ncbi-nlm-nih-gov.ezp.welch.jhmi.edu/pubmed/?term=Trelles%20M%5BAuthor%5D&cauthor=true&cauthor_uid=27810881). Emergency department care for trauma patients in settings of active conflict versus urban violence: all of the same calibre? [Int Health.](https://www-ncbi-nlm-nih-gov.ezp.welch.jhmi.edu/pubmed/27810881) 2016 Nov;8(6):390-397. Epub 2016 Nov 3.
